# Progress and challenges in developing organoids in farm animal species for the study of reproduction and their applications to reproductive biotechnologies

**DOI:** 10.1186/s13567-020-00891-w

**Published:** 2021-03-10

**Authors:** Guillaume Bourdon, Véronique Cadoret, Gilles Charpigny, Anne Couturier-Tarrade, Rozenn Dalbies-Tran, Maria-José Flores, Pascal Froment, Mariam Raliou, Karine Reynaud, Marie Saint-Dizier, Alice Jouneau

**Affiliations:** 1 INRAE, CNRS, Université de Tours, IFCE, PRC, 37380 Nouzilly, France; 2 CHU Bretonneau, Médecine et Biologie de la Reproduction-CECOS, 37044 Tours, France; 3 Université Paris-Saclay, UVSQ, INRAE, BREED, 78350 Jouy-en-Josas, France; 4 Ecole Nationale Vétérinaire D’Alfort, BREED, 94700 Maisons-Alfort, France; 5 Faculty of Sciences and Techniques, University of Tours, 37200 Tours, France

**Keywords:** Embryoid, Endometrium, Follicle, Organoid, Ovary, Oviduct, Testis, Placenta, Uterus

## Abstract

Within the past decades, major progress has been accomplished in isolating germ/stem/pluripotent cells, in refining culture medium and conditions and in establishing 3-dimensional culture systems, towards developing organoids for organs involved in reproduction in mice and to some extent in humans. Haploid male germ cells were generated in vitro from primordial germ cells. So were oocytes, with additional support from ovarian cells and subsequent follicle culture. Going on with the female reproductive tract, spherical oviduct organoids were obtained from adult stem/progenitor cells. Multicellular endometrial structures mimicking functional uterine glands were derived from endometrial cells. Trophoblastic stem cells were induced to form 3-dimensional syncytial-like structures and exhibited invasive properties, a crucial point for placentation. Finally, considering the embryo itself, pluripotent embryonic cells together with additional extra-embryonic cells, could self-organize into a blastoid, and eventually into a post-implantation-like embryo. Most of these accomplishments have yet to be reached in farm animals, but much effort is devoted towards this goal. Here, we review the progress and discuss the specific challenges of developing organoids for the study of reproductive biology in these species. We consider the use of such organoids in basic research to delineate the physiological mechanisms involved at each step of the reproductive process, or to understand how they are altered by environmental factors relevant to animal breeding. We evaluate their potential in reproduction of animals with a high genetic value, from a breeding point of view or in the context of preserving local breeds with limited headcounts.

## Introduction

The story of an individual begins with the fertilisation of an oocyte by a spermatozoon. Beforehand, formation of these mature and functional gametes from primordial germ cells (PGCs) relies on interactions between the germ cells themselves and somatic cells of various types within the gonads (ovary and testis) and, for spermatozoa, with the epithelial cells lining the lumen of the female genital tract. After fertilisation, the early embryo (zygote) undergoes cleavages as it progresses through the oviduct and uterus, where embryo–maternal interactions take place. When the embryo has developed into a blastocyst, it presents a cystic structure formed by the outer layer of the first differentiated cell lineage, the trophoblast, which encloses a group of cells called the inner cell mass. The trophoblast interacts with the uterus to prepare for implantation and will then give rise to the placenta. The inner cell mass contains the precursor of the pluripotent epiblast and of the primitive endoderm (or hypoblast). The epiblast will give rise during gastrulation to all somatic lineages as well as the germ cells. The primitive endoderm will form some extra-embryonic structures such as the yolk sac.

Physiological in vitro models of all these processes are invaluable in delineating the underlying mechanisms and in defining pertinent biomarkers of the viability and robustness of the biological structures or organs involved. They would help in the understanding of the causes of infertility or sub-fertility. Besides, they would have applications in assisted reproduction for humans and animals. In domestic species, especially in cattle, in vitro fertilisation and culture of embryos are widely used in commercial settings. Ultimately, organoids for reproductive organs would provide the optimal setting for culture and cryopreservation of gametes and embryos by mimicking their natural environment. In the field of animal breeding and preservation of animal biodiversity, mature gametes and embryos (embryoids) might be generated entirely in vitro. Organoids would also be useful in evaluating the long-term effect of environmental changes on the successive steps of the reproductive process. Nowadays, many environmental contaminants are a threat to human fertility, but also to farm animal fertility, as they can be found in grass, in animal food and even in the air. Climate change can also have an impact on reproductive ability. Overall, generating organoids of reproductive organs as well as embryoids will have many applications in the study of reproduction in farm animals. Among these, rabbit, pig, sheep and cow also constitute biomedical models of human reproduction, allowing both in vitro (in organoids) and in vivo studies. In this chapter, we will present the specific questions and the state of the art of organoid development related to female and male reproduction as well as embryoids.

## In vitro development of ovarian follicles for understanding ovarian function and improving assisted reproductive technologies

Organoids somehow contradict William Harvey’s quotation ‘*Ex ovo omnia*’, even more so when considering an ovarian organoid, which would turn the fertilisable egg from being the origin into being the target.

### Physiological background: formation and development of follicles, the functional units of the ovary

In vivo, a fertilisable oocyte able to sustain early embryo development results from a lifelong development. Briefly, from the female epiblast, PGCs are specified; they proliferate as they migrate to the genital ridge and form cysts of oogonia, which subsequently become oocytes arrested at meiotic prophase. Before or shortly after birth, oocytes recruit surrounding ovarian somatic cells to form structural and functional units called follicles. Primordial follicles continually leave the pool; the oocyte grows while its companion granulosa cells differentiate and proliferate to form several layers, and ovarian cells are recruited to form the theca at the rim. Then, a fluid-filled cavity, the antrum, forms. The growing antral follicle becomes dependent on gonadotropin hormones. Eventually, one or several follicles (in mono- or poly-ovulatory species, respectively) are selected to undergo final maturation: upon the luteinizing hormone surge, the enclosed oocyte resumes meiosis to progress to metaphase II before being ovulated in the oviduct. Folliculogenesis is controlled by the continuous dialogue between cellular compartments through the exchange of molecules and modulation of signalling pathways and by secretions from blood vessels (reviewed in [1]). In short, ovarian follicles are organised spherical scaffolds of three cell types (granulosa cells, theca cells and oocytes) of distinct origin. By producing fertilisable oocytes, they are the functional units of the ovary.

### Social and economic context

In humans, fertility preservation through ovarian cortex cryopreservation for future re-implantation has become widely proposed to patients about to undergo damaging chemotherapy treatments and meets increasing success [2]. However, for some patients, ovarian cortex re-implantation carries a risk of re-introducing malignant cells [3]. This has prompted the emergence of alternative biotechnologies, including the development of ovarian organoids, with the goal to achieve in vitro the completion of ovarian folliculogenesis, possibly including oogenesis from stem cells. In farm animals, these technologies could be implemented to increase the reproductive potential of individuals of high genetic value and therefore high economic interest. In particular, they carry the potential to generate offspring from prepubertal animals and reduce the inter-generation interval, to accelerate genetic progress and reduce breeding costs. They will be applied to maintain biodiversity through the preservation of local breeds with limited headcounts. Organoids could also be useful to improve reproduction in endangered species. Apart from assisted reproduction, in vitro systems are promising tools in toxicology to evaluate the damaging potential of drugs and environmental contaminants, particularly endocrine disruptors, on the ovarian pool of follicles and ovarian function. Finally, such an in vitro model of follicle development would be an invaluable tool to study and delineate the complex molecular dialogue between the oocyte and somatic cells that controls folliculogenesis.

### Ovarian organoids and in vitro follicle development in murine and human models

Multi-step systems combining ovarian explant culture with culture of isolated follicles have been successful at producing fertilisable eggs in the mouse. Culture of newborn ovaries was followed by isolation and further two-dimensional (2D) culture of oocyte/granulosa cell complexes and final oocyte maturation; the resulting mature eggs were inseminated and some of these generated live offspring after transfer into recipients [4, 5]. In the mouse, ovaries from foetus at 12.5 days of gestation contain PGCs. Starting from such immature cells, fertilisable and developmentally competent oocytes were produced in a similar but longer three-step culture system [6]. In a different approach, mouse embryonic stem cells (ESCs) were induced into PGC-like cells, which were then aggregated with E12.5 ovarian somatic cells to form follicle-like structures; following 2D culture, oocytes were matured and inseminated and could produce healthy pups [7, 8]. Thus, the rodent model provides proof of concept of an ‘artificial ovary’ by achieving complete oogenesis in vitro in less than 40 days, but the protocol requires supporting in-vivo-differentiated ovarian cells.

In humans, a related strategy attempted to generate oocytes and follicles from pluripotent stem cells. Human ESCs or iPSCs (induced pluripotent stem cells) were induced in vitro to differentiate into PGC-like cells [9, 10], or further into follicle-like cells, including a large central oocyte-like and small surrounding granulosa-like cells, which however failed to recapitulate the spherical follicle geometry [11]. Alternatively, putative oogonial stem cells collected from adult ovaries differentiated into haploid oocyte-like cells [12]. Building on previous progress in human follicle culture (reviewed in [13, 14]), human metaphase II oocytes were obtained over 21 days from large preantral follicles isolated from cultured ovarian pieces and 3D (3-dimensional)-cultured to the antral stage before the eventual 2D-culture and maturation of isolated oocyte–somatic complexes [15]. The ability of these oocytes to be fertilised and sustain early embryo development was not evaluated.

### Challenges and progress in farm animal species: biological issues to be addressed with 3D models of folliculogenesis

Compared to the mouse model, folliculogenesis in farm animal species presents major challenges. One is related to the duration of the process in vivo, estimated at 24 days in mice [16] but at least 4 to 6 months in ruminants, including 20 to 40 days after antrum formation. A second impediment is the size of antral follicles, which reach, at ovulation, 7 mm in the ewe, 20 mm in the cow and up to 45 mm in the mare, although some oocytes collected from smaller follicles (2–3 mm in ewe, 4–5 mm in cow) can sustain early embryo development in vitro [17].

**Figure 1:**
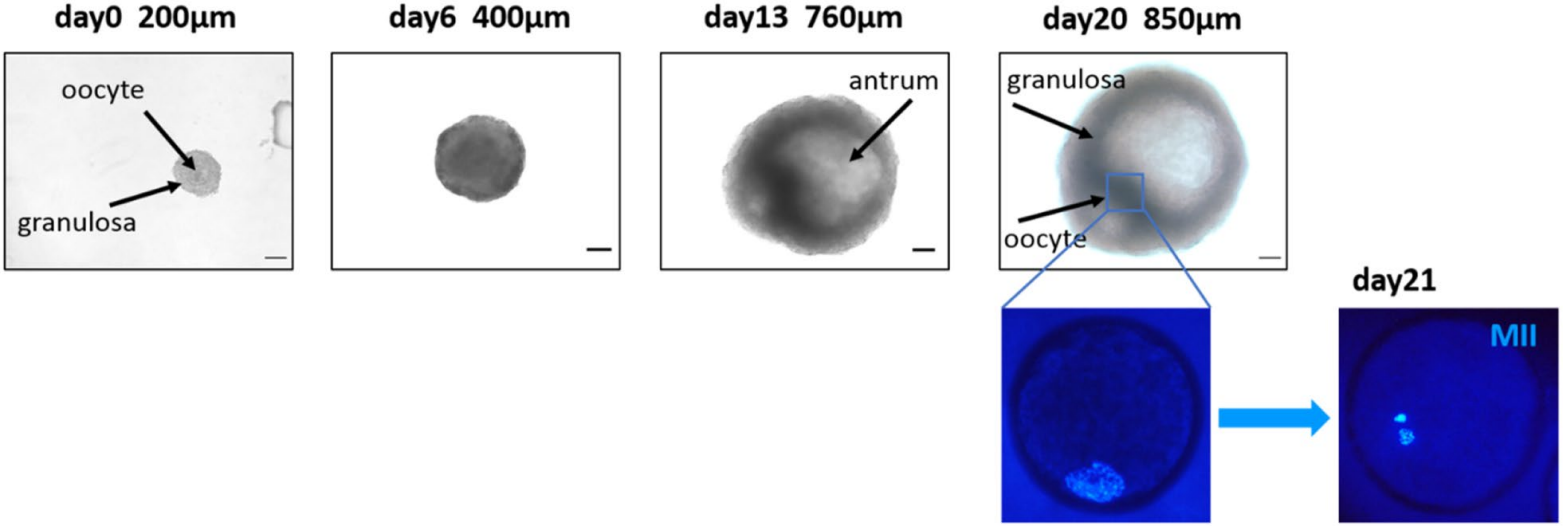
**Generation of a mature oocyte from a preantral ovine follicle.** The preantral follicle was isolated from the ovary and photographed after 6, 13 and 20 days of culture. Antrum formation was clearly observed from Day 13 onwards. The oocyte with surrounding somatic cells was extracted from the follicle and resumed meiosis up to the Metaphase II (MII) stage on the next day. Scale bar: 100 µm.

Considering the lack of conclusive studies on the culture of isolated primordial and primary follicles (in ovine species, [18, 19]; in bovine species, [20, 21]), most efforts have been devoted to obtaining competent oocytes from preantral follicles. From this stage onwards, the spheroid shape of the follicle is critical to its survival and growth, and therefore designing an adequate sequential 3D-culture system for several weeks appears crucial (reviewed in [22, 23]). Isolated preantral follicles were successfully 3D cultured in suspension beyond antrum formation in cow [24, 25], ewe [26–28], goat [29, 30] and sow [31]. Including follicles in an alginate-based matrix could be beneficial if adapting the compressive force to follicle expansion [22, 30]. Many studies have sought to improve the composition of the culture medium (by adding hormones or growth factors) of ruminant preantral follicles, whose quality and functionality were evaluated mainly based on their survival and growth, antrum formation, oestradiol secretion and/or the ability of the enclosed oocyte to undergo meiotic maturation (ewe [26, 27, 32], goat [30, 33]). Some oocytes collected from such in vitro grown follicles were able to sustain embryo development up to the morula stage (day5–6) (buffalo [34], ewe [35, 36], goat [37] and sow [38]). In our own system, the transcriptome of granulosa cells from cultured sheep follicles showed that they expressed markers of differentiation at a smaller follicle diameter compared to their in vivo counterparts; they also secreted more oestradiol and less anti-mullerian hormone, confirming that follicle development was accelerated in vitro compared to their in vivo counterparts [27]*.* Starting from 200-µm preantral follicles, our system allowed to obtain 30% meiotically competent oocytes from follicles as small as 600–900 µm in diameter in 20 days (Figure 1); this system may thus allow a decrease in the anticipated culture time of antral follicles in sheep and to limit the technical difficulties in culturing larger spherical structures. The system now needs to be further improved through supplementation with growth factors and possibly in a matrix maintaining an optimised spherical structure and dialogue between the oocyte and somatic follicular cells.

### Perspectives and conclusion on ovarian organoids

Ovarian follicle development determines oocyte quality, and therefore, knowledge of the molecular mechanisms controlling follicle dynamics is crucial for better identifying causes of infertility. Extensive research on follicular development in vitro has been conducted, both as a culture model for understanding the biological process itself and as a tool to improve assisted reproductive technologies in humans and animal species. Impressive progress has been made within the past decades, yet in non-rodent species these culture systems remain suboptimal, as a minimal proportion of cultured follicles produce oocytes capable of developing into blastocysts, and further into offspring, which is the ultimate functional criterion for evaluating an ovarian organoid.

## Development of testicular organoids to decipher the in vitro germ cell environment

The testis produces spermatozoids and synthesises androgens. It is a complex organ composed of different cell types creating a minimal structure called the seminiferous tubule. This tubule is composed of germ cells, peritubular cells and polarised Sertoli cells needed for germ cell differentiation. Between the seminiferous tubules, the interstitial space contains Leydig cells (producing testosterone) and vasculature. Like the brain, the testis has a defence against external aggression, which is the blood–testis barrier. This barrier creates a protected environment for germ cells against infection, toxic exposure or stress. It is able to ensure the translocation of germ cells from the basal pole to the apical pole but also to guarantee a physico-chemical and immunological barrier [39].

### Social and economic context

The deterioration of the male reproductive function has become of societal and agronomical concern and raises questions about the link between environment quality, breeding methods and the consequences of selection on testis function. To study infertility and/or testicular functions, the development of new in vitro strategies has opened up new areas in complex organ analysis, reprotoxicology and regenerative technologies, with the arrival of the testicular organoid concept [39–41].

### In vitro models: 2D culture

One of the first approaches applied was a germ-cell culture or co-culture of human germ cells on a monolayer of feeder cells in a 2D manner using standard culture dishes. In the 1960s to 1980s, the team of Dr Steinberger and Dr Toppari demonstrated the importance of temperature (close to the scrotum, i.e., 32 °C) and hormones in the survival, proliferation and differentiation of germ cells making spermatogenesis possible in in vitro cultures [42]. Several improvements to the culture methodology were made in the medium composition to provide both testicular tubulogenesis and spermatogenesis in vitro [43, 44], but the spatial arrangement of testicular cells was not reproduced, with negative consequences on spermatogenesis. The use of permeable supports (inserts) or three-dimensional (3D) cultures using artificial extracellular matrixes was a big step forward in the 1980s. Indeed, Hadley's work highlighted the role of extracellular matrixes to improve the 3D architecture of Sertoli cells, ensuring cell polarisation with tight junctions in the basolateral position, similar to in vivo [45, 46]. With inserts, several studies in rat and humans have succeeded in reproducing the complete process of meiosis and the differentiation of germ cells into haploid cells (spermatids), despite low efficiency (about 2% of cells were haploid cells) [47–49].

### Current models of testicular organoids

Testicular organoids have so far been realised in rodents, pigs and humans. Despite the diversity of cell types in the testis, most testicular cells can be derived from iPSCs, including PGCs or spermatogonial stem cells, which can self-renew and differentiate into the testicular organoid. The purification of these cells have revolutionised this field [50–52].

The medium used for most of the testicular organoids in rat and human species contained 10% KnockOut serum replacement, insulin and Vitamin A in order to develop the testicular tubulogenesis. A variety of artificial extracellular matrixes has been tested to favour the development of seminiferous tubules in vitro. Three different organoid protocols have been developed and are described below.

**Figure 2:**
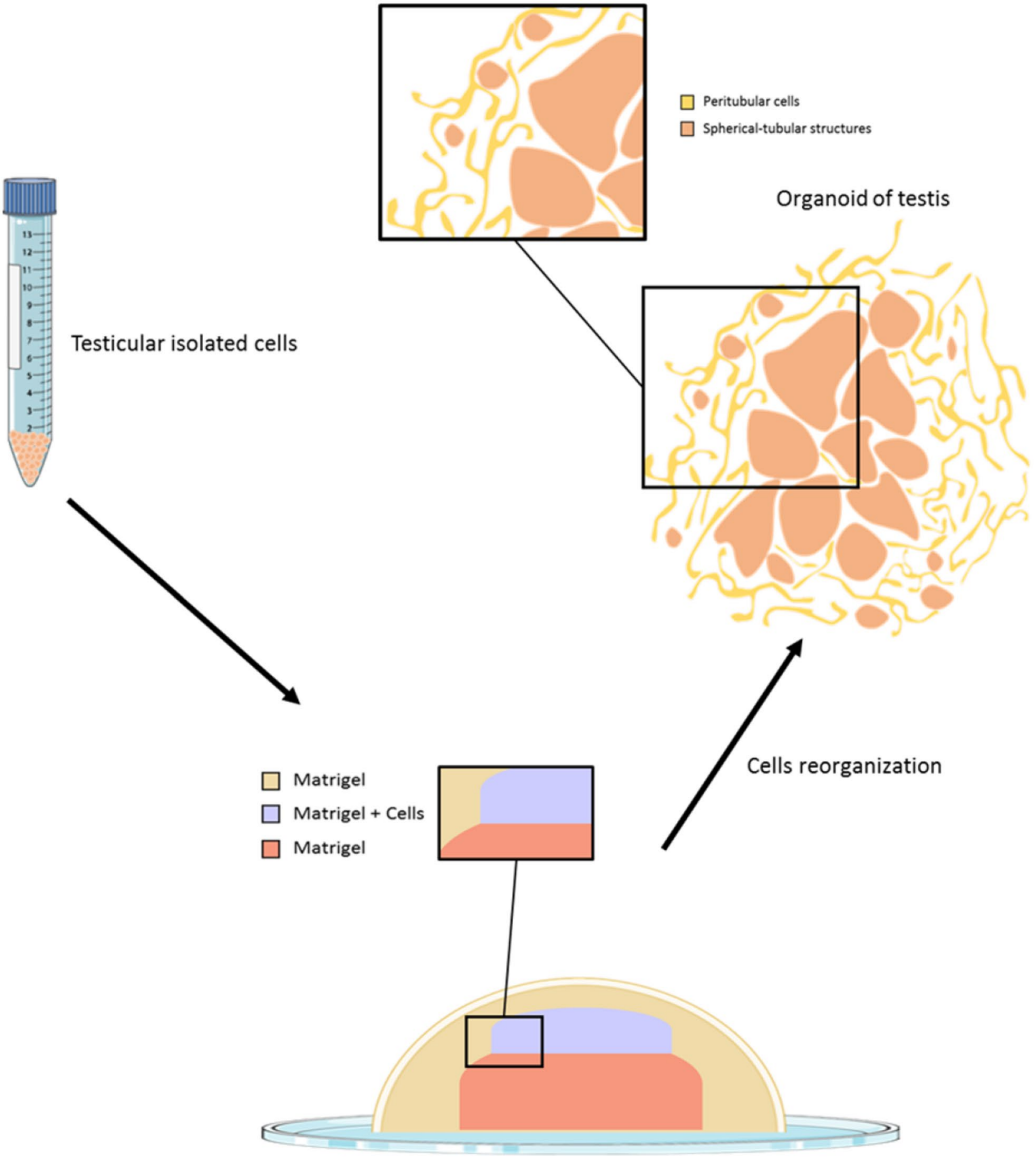
**A schematic representation of testicular organoid culture.**

Testicular cells from 18-day-old rat were cultured using inserts as follows: on the bottom of the insert, peritubular cells were cultured, mimicking the basal membrane, and on the top, Sertoli and germ cells were embedded within Matrigel^©^. The testicular organoid presents a cord-like structure with the presence of a blood–testis barrier. Polarised Sertoli cells favour the migration and maturation of germ cells to the centre of the structure [53]. The use of a culture medium flow mimicking blood flow improved oxygen, nutrient and cell viability compared to static organ-cultures [54]. In another study, rat prepubertal primary testicular cells were cultured in three concentric drops of Matrigel^©^ (Figure 2). This mix of germ, Sertoli and peritubular cells was able to rebuild tubule-like structures. The system of three-layer gradient of Matrigel^©^ permits the generation of functional organoids with a blood–testis barrier and proliferation of spermatogonial stem cells [55, 56]. Recently, in porcine, a testicular organoid model from 1-week old testicular cells was generated using a microwell centrifugal aggregation system [57].

The third model was the development of the first human testicular organoid. For that, a mix of immortalised somatic cells and primary adult human germ cells was used. The human Sertoli and Leydig cells were immortalised by overexpression of hTERT (Human Telomerase Reverse Transcriptase) after lentiviral infection. Then, Sertoli cells, Leydig cells and spermatogonial stem cells were suspended in a testicular organoid medium composed of solubilised human testis extracellular matrix, foetal bovine serum in StemPro-34 medium, a medium specifically formulated to support progenitor cells. All cells were cultured as hanging drops. Despite alteration of the architecture and tubule structure, probably due to differences between immortalised and primary cells, the human testis organoid was able to survive for up to 21 days and produced androgens. Expression of marker of haploid germ cells, PRM1 and Acrosin genes suggest that spermatogonial stem cells were able to differentiate into round spermatids in this first human testicular organoid [58, 59].

The development of tubulogenesis has been refined by modifying the composition of the extracellular matrix. Indeed, the matrix is specific to the testis and the development of a decellularised testicular matrix by digesting with sodium dodecyl sulfate detergent is able to improve the optimal scaffold for recellularisation with testicular cells and a better 3D organisation [40].

### Biological issues to be addressed by testicular organoids in domestic species

The development of testicular organoids is an alternative of choice for domestic species, but also for wild species, to evaluate the reprotoxic effects of molecules present in the environment (including food), or to explore environmental effects such as elevation of temperature and ground and water quality. Indeed, Prof. Habert has demonstrated that each species presents a different sensitivity to xenobiotics, and rodents are not always a suitable model, as has been reported for phthalate, whose effects on steroid production are largely species-specific [60].

Moreover, organoids are a good tool for germ cell conservation. In birds for example, embryo cryopreservation is not feasible due to the high lipid content in the egg, and chicken semen conservation has variable success depending on the breed. It is also a promising tool for genetic rescue of endangered and wild species [61]. Some species could be saved through male germ cells and germinal stem cells as a way to regenerate gametes [62]. Generation of spermatids using organoids is also of interest for species with high-value specimens such as horses, where intracytoplasmic sperm injection has become a useful technique in the horse-breeding industry.

Testicular organoids would be of interest as a biomedical model for humans. Indeed, in the case of anti-cancer treatment in humans, the germ cells are destroyed. The only fertility preservation option for young boys who are not able to cryopreserve sperm is to conserve and culture testicular tissues that contain spermatogonial stem cells. Other options would be the reinjection of these spermatogonial stem cells into the testis after treatment or to induce germ cell maturation in vitro followed by intracytoplasmic sperm injection.

Several perspectives in domestic species will stimulate the development of organoid culture mimicking in vivo spermatogenesis towards reprotoxicity assay, management of livestock of interest, protection of endangered species and development of regenerative medicine. Finally, the development of these approaches can help to decipher certain pathological situations. For example, metabolic diseases such as diabetes, which has significant societal consequences, impact the production and quality of spermatozoa, and a better understanding of the altered mechanisms is necessary. Moreover, the genetic selection of domestic animals such as poultry has led to a drop in their fertility, and a better knowledge of the affected pathways could be helpful in new breeding and genetic strategies.

## Three-dimensional models of the oviduct for the study of the periconception period and improvement of assisted reproductive technologies

The oviduct hosts crucial steps for the reproductive success: sperm storage before ovulation, capture of the ovulated ovum, acquisition by sperm of fertilising competence (capacitation), fertilisation, and the first steps of embryo development, all take place in the oviduct, known as the fallopian tube in humans. The oviduct is highly sensitive to its maternal environment, leading to fertilisation failure in cases of abnormal hormonal environment or oxidative or heat stress [63]. Up to 50% of pregnancy failures in dairy cattle take place in the oviduct [64], highlighting the potential impact of this first interface between the mother and the early-developing embryo. The oviduct epithelium is a simple, columnar shaped, highly polarised epithelium composed of ciliated and non-ciliated cells. However, oviducts are small intra-abdominal organs not accessible in vivo without surgery or slaughter, making it difficult to study their physiology. Contrary to common belief, the oviduct is not a plain, biologically inert duct but is lined with a convoluted mucosa presenting numerous folds, pockets and cul-de-sacs [65]. The oviductal lumen is filled with microliters of highly complex and regulated secretions [66], offering a challenge for the development of physiological in vitro models. Classical 2D in vitro models using primary culture of oviduct epithelial cells (OECs) have been used extensively to investigate the interactions between gametes/embryo and oviductal cells. However, OECs grown on plastic dishes dedifferentiate rapidly, lose their cilia and the ability to respond to steroid hormones, and secrete specific oviductal markers [67]. Alternative in vitro models able to maintain the secretions and morphology of OECs have been successfully developed and are still in progress.

### Developed 3D models and organoids derived from oviductal cells in mammals

**Figure 3:**
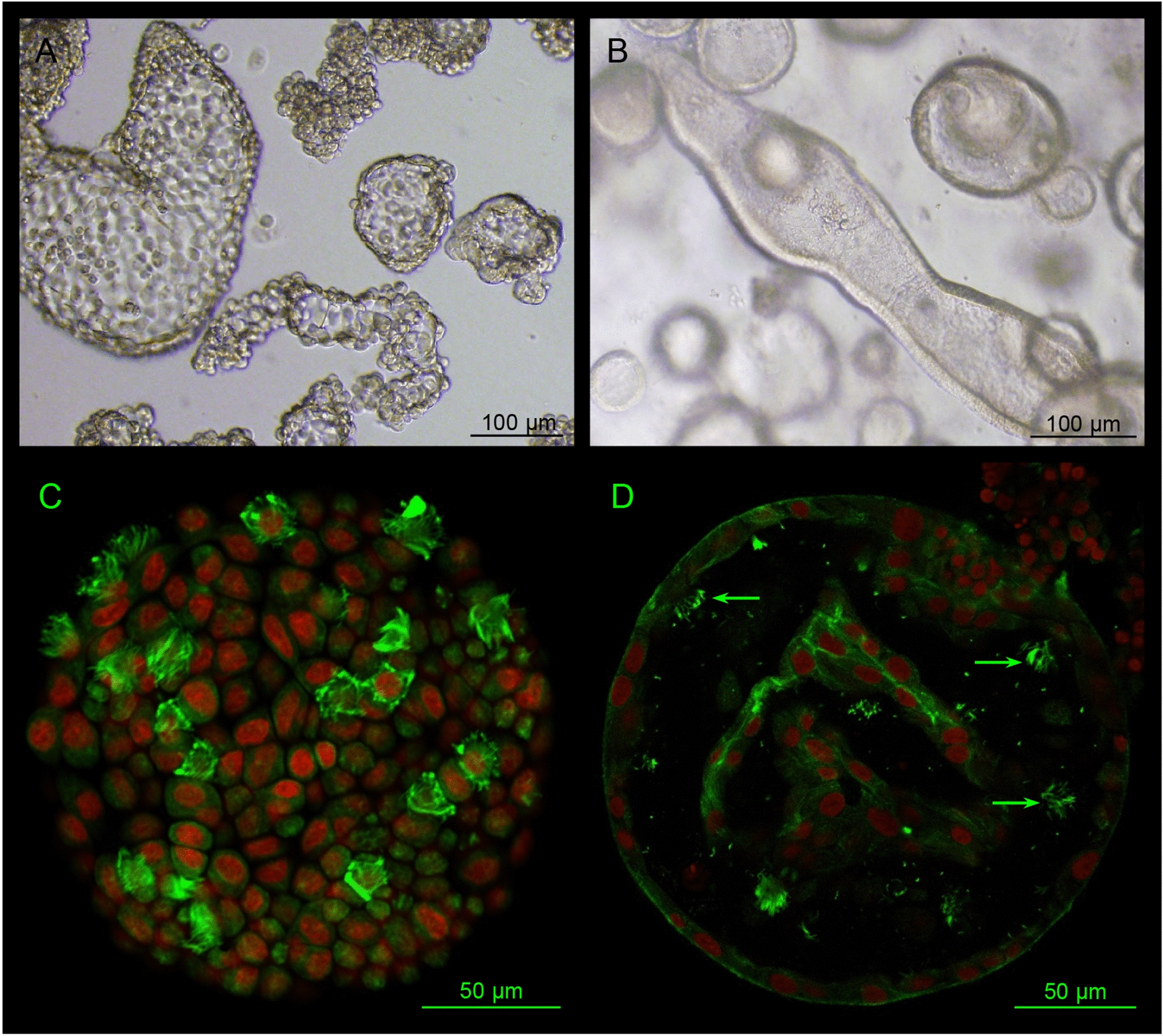
**Bovine oviduct 3-D models.** (**A**) Oviduct aggregates/multicellular vesicles derived from primary cultures of bovine oviduct epithelial cells at day 10 of culture; (**B**) Oviduct organoids derived from bovine oviduct stem/progenitor cells at day 20 of culture; (**C**, **D**) Confocal microscopy pictures showing the presence of both ciliated (with alpha-tubulin in green) and non-ciliated cells (nuclei in red) in one vesicle (**C**) and one spherical organoid (**D**). Note that the apical end of cells, with cilia, is oriented outside in vesicles and towards the lumen in organoids.

Sheets of bovine oviductal mucosa placed in a culture medium spontaneously form floating aggregates and multicellular vesicles of various sizes (100–1000 µm) containing a mix of well-polarised ciliated and non-ciliated secretory cells that are normally present in the oviduct epithelium, with their apical side oriented outside [68]. The time during which OECs within these vesicles can remain fully differentiated has not been reported. Another strategy used successfully for long-term culture of differentiated OECs in mice, bovine and porcine consist of culturing OECs on inserts in an air–liquid interface: growth medium is provided to the basal side of the cells while the medium is removed from the apical side, allowing the cells to differentiate morphologically and functionally over several weeks [69]. In addition, a 3D oviduct-on-a-chip model in a two-compartment microfluidic device has been recently reported in bovine, where a layer of confluent OECs was grown on a porous membrane in the upper compartment while circulating hormonal changes were mimicked in the basolateral compartment [70]. In these models made using primary cell cultures, in-vivo-like morphological characteristics of OECs could be maintained for up to 12–14 days, making them suitable to study in vitro sperm–oviduct and embryo–oviduct interactions [68, 70, 71]. However, OECs in primary cultures end up dedifferentiating [72], so that repeated collections at a slaughterhouse are needed to regularly start new cultures. The existence of adult stem/progenitor cells (ASCs) has been reported in the oviduct of humans [73] and mice [74]. In both species, spherical oviduct organoids composed of fully differentiated ciliated and secretory OECs (with their apical side towards the lumen) have been successfully generated from sorted and Matrigel^©^-embedded ASCs [73, 75, 76]. Oviduct organoids can reproduce the in-vivo-like folds of the oviduct epithelium and can be maintained long-term (> 16 months) in culture [73]. Of note is that the presence and characterisation of oviductal ASCs has not yet been reported in farm animals. However, these progenitor cells are likely to be present for the regeneration and repair of the oviduct epithelium in these species [77]. Furthermore, bovine oviduct organoids have been recently obtained at INRAE from isolated OECs (preliminary unpublished data; Figure 3), showing that oviductal organoids can be generated in farm mammalian species and opening a new perspective for fundamental and biotechnological research.

### Biological issues to be addressed with 3D models of oviductal functions in farm animals

Oviductal organoids in humans and mice have mostly been used as models to study the origin and development of epithelial ovarian cancers, as the fallopian tube has been implicated as a site of origin of aggressive cancers [73, 75, 76]. In farm animal species, new physiological in vitro 3D models of the oviductal epithelium are needed to understand better the action of systemic regulators (e.g., ovarian steroid hormones) and local cellular interactions with gametes and embryos during the periconception period.

In most female farm mammals, the oestrous cycle is on average 21 days, i.e., much longer than the 4–5-day cycle in mice and very different from the menstrual cycle in terms of hormonal regulation. The oestrous cycle typically contains a short (2–3 days) follicular phase and a long (18–19 days) luteal phase with fluctuating circulating levels of the ovarian steroid hormones progesterone and oestradiol. Organoids can be cultured long-term in presence of hormones and, as such, may constitute valuable models to examine the morphological and physiological changes of the oviductal epithelium and its secretions throughout the oestrous cycle.

The improvement of assisted reproductive technologies (ARTs) in farm animals appears crucial to meet the challenges of livestock production and to preserve the diversity of domestic species. However, in vitro conditions for gamete storage, fertilisation and embryo production in animals are still far from optimal. Polyspermy is a frequent phenomenon during in vitro fertilisation in many species, including pigs, while it is rarely observed in vivo [78]. Furthermore, embryos produced in vitro are substantially different from their in vivo counterparts in terms of molecular composition and epigenetic reprogramming. This is associated with a reduced likelihood of pregnancy after transfer to a recipient mother and with increased health issues after birth [79]. The use of more physiological 3D oviduct models may help to understand the cross-talk taking place between the mother and the gamete/embryo leading to the establishment of pregnancy. It may thus contribute to improvements in the field of assisted reproduction for animals and humans. To illustrate, microfluidic and 3D devices have been proposed to improve the selection and capacitation of DNA-intact spermatozoa in pigs [78]. The 3D oviduct-on-chip model mentioned above supported more physiological genetic reprogramming than conventional in vitro fertilisation in produced bovine zygotes [70]. Oviduct organoids, combined with microinjection of gametes or embryos inside their luminal compartment, may represent the most physiological model since they allow reproduction of the folded tubular structure of the oviduct and concentrate in-vivo-like secretions in their luminal compartment.

## Endometrial organoids as a model for maternal–conceptus interactions and reproductive disease in cattle

The endometrium is not, strictly speaking, a morphologically complex tissue, but this organ is highly complicated from a functional point of view. It undergoes a dynamic change at each sexual or oestrous cycle depending on steroid hormones and also has to remodel in response to embryo implantation and pregnancy processes. The mammalian endometrium is comprised mainly of two main cellular types: epithelial cells and stromal cells. The endometrial epithelial tissue is organised into two different structures: the glands and a monolayered luminal epithelium lining the cavity of the uterus. Endometrial glands are tubular glands invaginating into the stroma and forming tubular structures that coil and branch more or less depending on the species [80, 81].

The idea that uterine secretions are essential for embryo development and pregnancy is an old and commonly accepted paradigm that has long been founded on empirical and indirect evidence. Based on the invalidation of gland formation, definitive evidence that uterine gland secretions are required to support pregnancy has been provided in mice and ewes [82, 83]. It is stated that any changes in the composition of uterine factors or in the precise adjustment in time and space of this production disrupt the functionality of the uterus in terms of supporting embryonic development and pregnancy. In fact, it is now admitted that the endometrium is a biological sensor able to fine-tune its physiology in response to the presence of individual embryos [84, 85]. Recruitment and accumulation of immune cells (monocytes, macrophages, and Dendritic Cells) in the gravid endometrium in cows [86] and ewes [87] are believed to be involved in elimination of apoptotic cells, immunosuppression, development of the conceptus and detachment of the placenta at parturition [88]. Thus, to avoid rejection of the embryo, the endometrium adapts a tolerance mechanism by secreting cytokines, growth factors and chemokines to create an inflammatory and immune environment propitious to embryo implantation [89] and to prevent infection [90]. In response to bacterial infection, endometrial cells activate the synthesis of pro-inflammatory cytokines. These cytokines and more specifically, TNFα promotes the migration of specialized immune cells from peripheral blood to the genital tract. This induces a rapid and robust innate immune response from endometrial cells through the production of interleukins (IL)1β and IL6, and chemokines, such as IL8, and acute-phase proteins [90].

### Biological issues to be addressed by endometrium organoids in livestock

In humans, understanding the causes of chronic endometrial inflammation, i.e., endometriosis, as well as treatment of endometrial cancer, justifies the involvement of many teams in the development of endometrial cellular models. In addition, the treatment of infertility is one of the main reasons for endometrial research in both animals and humans. In ruminant species, an inadequate dialogue process between the developing embryo and the maternal organism results in embryo losses within the first three weeks after fertilisation [64]. One first cause of embryonic mortality is related to improper endocrine control of the endometrial function involved in embryo development and uterine receptivity. In vivo studies have been able only very partially to describe the interrelationships between the maternal organism and the embryo in early gestation. It is hoped that the use of organoids will help to overcome the insufficient current knowledge on the control of embryo development by uterine molecules and to go further in the understanding of mechanisms of early gestation. The first objective of using endometrial organoids is to describe the processes controlling the synthesis and secretion of uterine factors. The second is to understand how endometrial factors control the development of the embryo and how the embryo, in turn, regulates the production of uterine factors. The combination of endometrial organoids and embryos in an organ-on-a-chip system will help to describe the dynamic biological processes in the dialogue between mother and embryo. The most important challenge of this project will be to achieve the successful implantation of embryonic trophoblastic tissues onto organoid cells.

Multipathogenic bacterial infections of the genital tract after calving disrupt the physiological events in many dairy cattle, leading to the development of uterine inflammatory diseases (retained foetal membranes, metritis and endometritis). These uterine pathologies are the second most important cause of alterations in the embryo–maternal interactions necessary for the establishment of pregnancy. The resulting uterine inflammatory diseases cause sub-fertility, delay conception, alter ovarian function and cause early embryonic mortality [91–94]. Their impacts amount to €1.4 billion per year for dairy farming in Europe [95]. Endometrial organoids will be good tools to study (i) uterine pathologies, (ii) ageing and (iii) endometrial gland biology and to reduce the number of experimental animals. This approach may help to characterise new targeted therapeutic strategies such as mitigation procedures (including microvesicles). It will allow the development of viable uterine organoid biobanks for various forms of uterine diseases as tools for improving uterine receptivity in pathological conditions.

### Towards development of complex 3D structures of the endometrium

While endometrial cell co-culture could improve embryo development in assisted reproduction protocols, such in vitro cellular models have failed to obtain embryo development beyond the blastocyst hatching stage. Attempts to study the early events of embryo implantation could not be properly achieved in any of co-culture systems used. The improvement of these cellular models has been a major concern of reproductive biologists and has been carried out in several ways.

In many mammalian species, monolayer cultures of both endometrial cell types have been established and the cells characterised with respect to their histochemistry and functional properties. Endometrial cells in primary culture or in cell lines have provided useful in vitro model systems for the study of hormone and cytokine action, signal transduction pathways, cell–cell interactions and gene expression in specific endometrial cell types.

The development of multicellular endometrial structures incorporating endometrial epithelial and stromal cells has long been in use. In 1986, endometrial spheroids structurally and functionally close to the uterine glands were proposed for the study of endometrial physiology [96]. Improvements in cell culture procedures, such as the stimulation of collagen production by stromal cells, have been used to promote the anchoring of epithelial cells and allow the development of spheroid structures, mimicking functional uterine glands [97]. Also, advances in tissue engineering have enabled the association of epithelial and stromal cells within a polymeric fibrous matrix mimicking the 3D structure of endometrial tissue [98].

Recently, two independent groups have proposed a method to establish long-term culture of organoids of human and mouse endometrium using endometrial cells [99, 100]. Although they were originally produced with a mixture of the two main endometrial cell types, the multiple generations of organoids produced were spherical structures consisting only of epithelial tissue. Since then, attempts have been made to generate more complex human endometrium organoids associating stromal and epithelial cells [101, 102]. Using a porous collagen scaffold, these organoids, which respect the interactions between the two cell types, recapitulate the tissue architecture and promise to be better tools for modelling the physiology of the endometrium. While human endometrial stem cells were evidenced in 2004 [103], it is only recently that the different endometrial cell types have been obtained from mesenchymal stem cell derivation. Following isolation of CD146 + cells by magnetic purification from an endometrial cell mixture, Fayazi and colleagues were able to form structures containing both epithelial and stromal cells, expressing the genes known to be specifically associated with each endometrial cell type [104].

In bovine, we have used primary endometrial epithelial cells isolated from uterus at Days 12–15 of the oestrous cycle. An approach based on hydrogel technology, in particular an extracellular matrix protein, was adapted from the human endometrium organoid protocol [100]. Our first results indicated that endometrial epithelial cells created spheroid structure. Using electron microscopy, detailed characterisation revealed polarised epithelial cells exhibiting tubular structure, with apical side outwards. Immunohistochemical staining revealed that the spheroids/organoids formed from endometrial epithelial cells express cytokeratin (preliminary results).

### Challenges for the development of endometrial organoids in livestock species

In mammals, the morphogenesis of the genital tract and particularly development of the uterine gland (adenogenesis) takes place under the effect of ovarian hormones during the postnatal period (for review see [105]). In addition, after calving, regeneration of the epithelium is complete after about 3 weeks. Thus, it has been suggested that resident or migrating uterine stem/progenitor cells participate in endometrial regeneration to restore tissue homeostasis [106]. In 2008, Donofrio and colleagues isolated for the first time from bovine endometrium primary cell lines displaying the functional characteristics of mesenchymal stem cells (MSCs) [107]. Several laboratories have shown that the endometrial compartment of the uterus is the major site of uterine stem cells in cows [108, 109]. It has been postulated that MSCs play a crucial role in stromal regeneration and differentiation during reproductive cycles and pregnancy [107]. With regard to their efficient isolation from endometrial biopsies [106], their plasticity and their capacity for self-renewal, could MSCs be candidates for the derivation of endometrial organoids? For this, it would be necessary to differentiate MSCs into epithelial cells, which constitute one of the major components of endometrial tissue. To date, under the standard culture conditions used in several laboratories, MSCs have been differentiated only into chondrogenic, osteogenic and adipogenic lineages [109] and into vascular smooth muscle cells [110]. A potential way to address this issue would be to use cellular reprogramming strategies to differentiate MSCs in the endodermal cells (epithelial cells) to derive endometrial organoids.

At last, another challenge will be to incorporate immune cells such as macrophages and T cells within endometrial organoids. This would greatly contribute to the understanding of the factors that determine an inflammatory immune response or the presence of the conceptus. The responses of these immune cells in co-culture with organoids could be evaluated by cytokine production in the presence of IFNT, progesterone or inflammatory agents (LPS). Until now, organoids have been developed by successive derivations of endometrial tissue samples. If immune cells can indeed be embedded in the original sample, these immune cells will not be able to survive after successive in vitro derivations of organoids. The methods will have to be adapted to include in the organoids immune cells taken from another animal under different physiological or pathological conditions*.* A platform using co-culture models of autologous tumor organoids and peripheral blood lymphocytes from colorectal cancer patients has been used to assess the sensitivity of tumor cells to T cell [111]. The organoid and immune cell co-culture models used in these studies could be adapted to endometrial organoids.

Despite current limitations, endometrium organoids will offer a promising platform to address fundamental questions regarding the reproductive system’s physiology involved in embryo development and differentiation. By combining endometrial organoids and embryos produced in vitro, organ-on-a-chip systems will be the most appropriate technology to deal with these issues. One of the main challenges will be to find the conditions to cultivate embryos and organoids simultaneously while preserving the functional integrity of both parts. Embryos are generally produced in an atmosphere of 5% O_2_, while organoids are cultivated at 20% O_2_. Since it has been demonstrated that reciprocal interactions between endometrial epithelial and the stromal cells are responsible for the uterine physiological functions, it is imperative that the organoids must associate these two types of cells. A second challenge will be to allow embryos access to histotrophic secretions. Histotrophs are molecules secreted in the uterine lumen in the vicinity of the embryo. However, accessing the secretion from the apical organoid surface is challenging because, in most cases, this surface is enclosed within the spheroid. It will be necessary to find a way to reverse the polarity of the organoids so that the apical surface faces the medium surrounding the embryo. Finally, an adequate microfluidics device will have to be developed.

## Models of embryonic development: blastoids and embryoids

In the mouse, three types of stem cells can be derived: pluripotent ESCs, trophoblast stem cells (TSCs) and extra-embryonic endoderm cells (XEN). These stem cells have revealed remarkable properties of self-assembly and self-organisation, allowing the development of models of the early development from blastocyst to post-implantation [112]. The field has also benefitted from recent optimisation of embryo culture beyond the blastocyst stage up to early implantation [113–115]. Two models have been developed in mice. First, by combining mouse ESCs and TSCs in non-adherent aggregates, blastocyst-like structures, named blastoids, eventually develop [116]. These structures have morphological and molecular features close to the blastocysts and some can even implant, although they resorb soon after. Second, using different culture media, these stem cells, along with XEN cells, can be aggregated in order to reconstitute an early post-implantation embryo [117–119]. In humans, ESCs or iPSCs have been shown to self-organise under controlled conditions into a cyst composed of an epiblast with its amnion, mimicking the morphogenesis of the human embryo after implantation [120, 121]. Moreover, mouse ESCs have an inherent plasticity that allows them to expand their potential by manipulating the signalling pathways and the culture medium. Such expanded pluripotent stem cells (EPSCs), potentially with TSCs, have generated blastoids able to develop up to a stage equivalent to early post-implantation embryo in the mouse [122, 123], thus combining both models.

### Biological issues to be addressed by embryoids in domestic species

**Figure 4:**
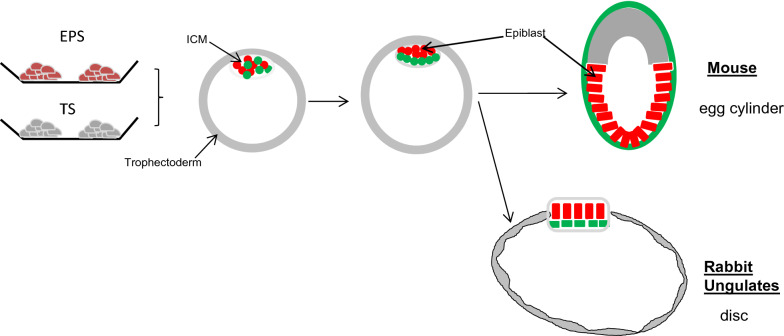
**Differences in development of the blastocyst in rodents and ungulates/rabbit.** The cup-shaped epiblast in the mouse develops after implantation, while in other mammals such as ungulates and rabbit, it develops as a disc, in contact with the uterine lumen, before implantation.

Although the development to blastocyst stage is very similar in several mammalian species, the subsequent development of the epiblast can be quite different [124]. In ungulates and in rabbit, in contrast to the mouse, the epithelialised epiblast develops as a disc that is open to the uterine lumen and connected only laterally to the trophoblast (Figure 4). This morphogenesis happens before implantation. Moreover, while the proliferation of the trophoblast is controlled by the underlying epiblast in mouse, in other mammals where contact between both tissues is reduced, this is less clear [125]. In such a context, generating blastoids and embryoids in these species would help greatly in deciphering the nature and role of the interactions between the epiblast and trophoblast, which are still poorly understood. Besides, the trophoblast tissue is highly proliferative, especially in ungulates, and the mechanisms controlling such remarkably fast and directed expansion are not well understood; uterine secretions most probably play a key role in the process [126]. It is noteworthy that this period of blastocyst elongation and epiblast morphogenesis is a period of high rate of gestational loss in cattle [127]. Combining a model of bovine uterine endometrium organoid (see Section 5) with blastoids would allow study of the elongation process and the uterine–embryo interactions. In addition, in the search for early biomarkers of embryo viability such an integrated model would provide a physiological method to test the relevance of any biomarker.

### Challenges in domestic species:

Embryoid/blastoid formation requires the availability of pluripotent stem cells and TSCs, which is an issue in domestic species where the search for the equivalent of mouse ESCs is a long-lasting quest [128]. In rabbit, several pluripotent cells with various pluripotency states have been derived [129]. A recent publication has reported the derivation of bovine pluripotent cells [130]. The potential self-organisation ability of such cells remains to be tested in a 3D environment in vitro*.* In addition, a similar approach to that used to generate EPSCs in the mouse has now been applied to reprogram both human and porcine cells [131]. Whether such a method could be used in bovine remains to be tested. Moreover, the exact nature and potential of these cells is a matter of debate [132].

In bovine, porcine and rabbit, trophoblast cell lines have been derived from blastocysts [133–136]. They can be used to study placental development and function, as outlined in the next section. Besides, these TSCs, together with pluripotent cells, can be tested for their self-assembly ability, which can be considered as a readout of their faithful equivalency to embryonic cells.

## Organoid generation for the understanding of placental functions

### Introduction to placental function

**Figure 5:**
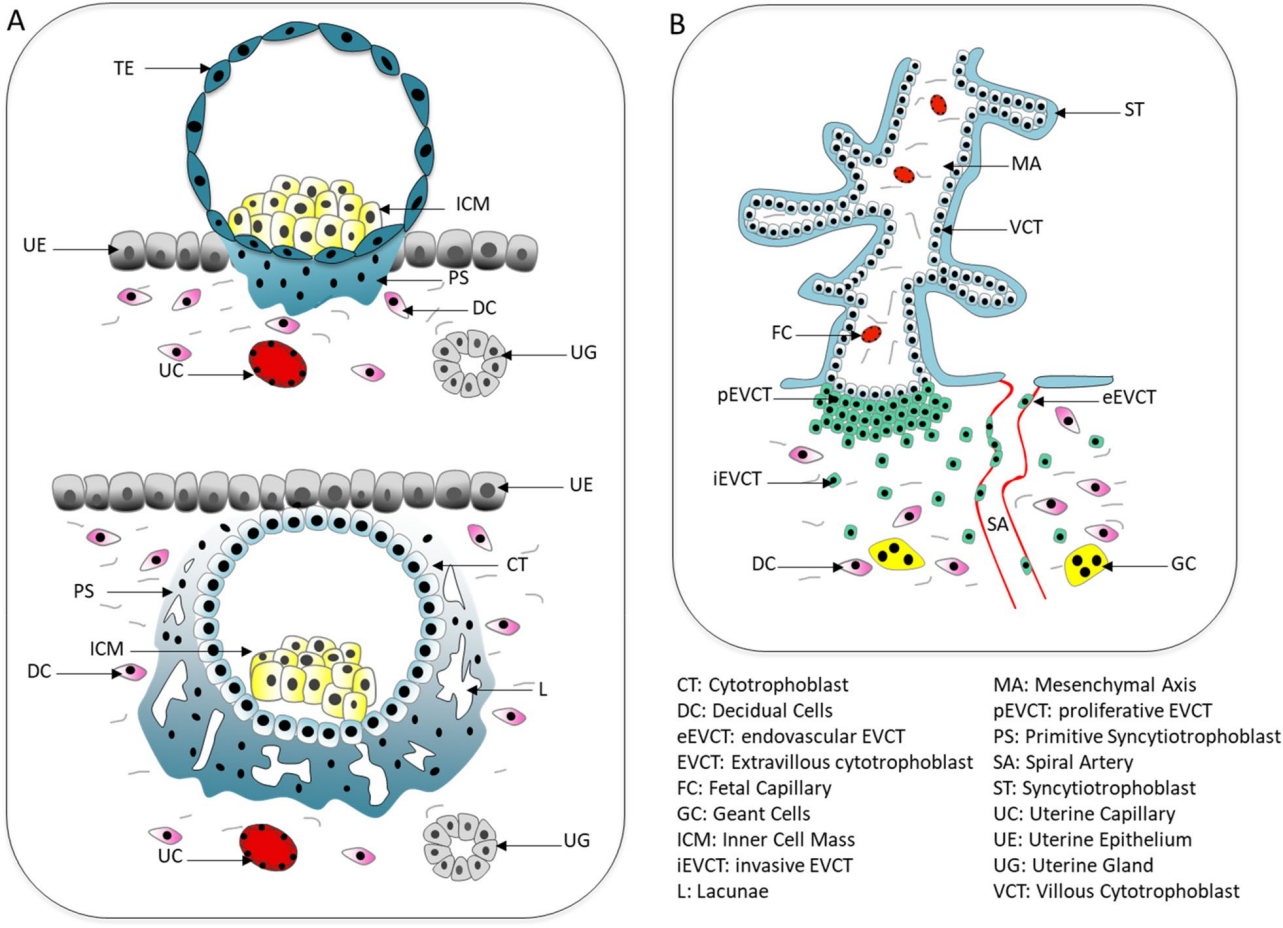
**Schematic representation of the anatomical and cellular structure of early-stage placentation**: **A** Blastocyst implantation and interaction with maternal endometrium; **B** Anchored foetal villi of the human placenta showing the extravillous trophoblast reaching the endometrium and the uterine spiral artery.

A trophoblastic cell lineage is essential for the survival of the embryo in utero. It plays a key role in the implantation of the embryo into the uterus and then forms most part of the placenta. This organ has several functions (see the schematic representation of early placentation in Figure 5). It induces immune tolerance to the allograft represented by the conceptus and ensures the transfer of nutrients, elimination of foetal waste and hormone production that will influence foetal, placental and maternal metabolism and therefore the development of the foeto-placental unit [137]. Due to its location, this organ is sensitive to the maternal environment, such as stress, nutrition, pollution, food and environmental contaminants [138–141]. These factors can affect its size, morphology, vascularisation, utero-placental blood flow, abundance of nutrient transporters, metabolism, hormone production, oxidative or nitrosative/NO stress, inflammatory status and molecular status (including epigenetic marks). Moreover, abnormal placentation can lead to various pregnancy complications, such as miscarriage, preeclampsia and intrauterine growth restriction, increasing the risk of metabolic syndrome in later life [142, 143].

### 2D and 3D trophoblast models

Trophoblast stem cells (TSCs) are the precursors of the differentiated cells of the placenta and are thought to exist in all placental mammals, especially during the early stages of placental development when trophoblast growth is maximal [144]. Mouse TSCs were the first to be established [145]. Other mammals TSCs have been derived since then, including monkey and human TSCs [146–148].

Monkey and human TSCs show a trophoblastic DNA methylation status as well as a trophoblastic gene expression profile and levels of miRNA corresponding to in vivo trophoblast cells. Moreover, they exhibit differentiation capacity towards both invasive trophoblast cells and multinucleated syncytia [146, 147, 149]. Monkey TSCs could also form syncytial-like structures and have demonstrated invasive capability similar to extravillous trophoblasts [148]. Extravillous trophoblasts invade decidual tissue to transform the spiral arteries at the first trimester, a crucial process for proper placentation [150].

A novel human placental barrier model has been developed consisting of the triple co-culture of hTSCs (derived from human induced pluripotent stem cells, hiPSCs), fibroblasts and umbilical vein endothelial cells. In this model, cells were able to form a syncytialised epithelium and express membrane transport proteins showing permeability results comparable to those found with ex vivo placenta [151].

Development of placental organoids has started recently, and they will provide a powerful tool for molecular and functional characterisation of trophoblast cells and in understanding the pathogenesis of developmental disorders associated with trophoblast defects. They will allow more complex studies on the early embryo/placental development or the response of the placenta to environmental factors. Attempts to obtain placental organoids have so far been successful only for human cells [152–154]. 3D trophoblast cystic-like structures were obtained from hiPSCs by a limited-area cell culture method with no chemical stimulation [153]. Haider and colleagues established human cytotrophoblast (CTB) organoid cultures from the first trimester (6–7 weeks of gestation). Villous CTBs were capable of self-renewal and expansion using a defined cocktail of factors (growth factors and signalling inhibitors). In these conditions, organoids were obtained after 2–3 weeks of culture and could be cryopreserved and recultivated; they could be expanded for more than 5 months, and transcriptomic analyses showed that they are similar to primary CTBs. However, no organoid could be generated from tissues derived from the tenth to twelfth week of gestation [152].

Other authors successfully generated long-term, genetically stable cultures of human trophoblast cells from placental tissue at 6–9 weeks of pregnancy that can differentiate into both syncytium and extravillous trophoblasts [154]. These organoids replicated the 3D structure of the placenta and were able to function for at least a year, still showing active mitochondrial function. They produced placental-specific peptides and hormones, including human chorionic gonadotrophin and pregnancy-specific glycoprotein. These organoids retain characteristic features of first trimester trophoblasts in vivo, as well as similar transcriptomic and global methylation profiles. Otherwise, by selecting the appropriate media both decidual glandular and trophoblast organoid cultures could be derived from the same placenta. This is, to our knowledge, the first research to demonstrate placental organoids lasting for over a year.

In summary, 3D trophoblast organoids are interesting tools to mimic trophoblastic differentiation and invasion capacities and to represent the in vivo structure of the placenta [152, 154, 155]. The establishment of 2D TSC cultures, as well as 3D trophoblast organoids, from abnormal placental tissues represents a future challenge to improve knowledge about pregnancy complications resulting from failures of trophoblast growth and/or differentiation.

### Trophoblast stem cells in domestic species

Concerning livestock, a serious problem faced by producers is the decrease in the conception rate of cattle, and reproductive failure is extremely costly. Embryonic losses occur mostly before Day 14 of pregnancy (time of elongation of trophoblast); hence, development and differentiation of trophoblast cells may affect conception ability in cattle [156].

Advances in TSC research in domestic species will help to understand and overcome this issue. Despite research efforts using the mouse, monkey and humans, the understanding of TSCs in other species, in particular in livestock species, has remained limited. Trophoblast cell lines have been derived from goat, cattle and pig [136, 157–160]. However, those cells are likely to represent a differentiation stage beyond that of TSCs, as stem cell characteristics have not been thoroughly examined.

Recently, bovine trophoblast stem-like cells similar in phenotype to mouse TSCs have been derived from in-vitro-produced blastocyst-stage embryos [161, 162]. Trophoblastic cell lines that possess trophoblast stem-like characteristics have also been generated for the first time after induction of pluripotency in bovine amniotic cells. They have the potential to differentiate into the extra-embryonic cell lineage [163]. These cell lines can be a model for basic research on peri-implantation and placental development in cattle and as donor cells for transgenic animal production [161, 163].

In our laboratory, we derived rabbit TSCs from blastocysts and differentiated them into cells that expressed both phenotypic characteristics and marker genes of trophoblast cells. They were able to differentiate into cytotrophoblasts and syncytiotrophoblasts onto a collagen gel in the presence of a flow of culture medium that mimicked maternal blood flow [134]. Among experimental animals, the rabbit has a placental structure closer to that of human placenta. The rabbit TSC culture model is close to primary cells and structurally more pertinent as a model for human placental studies than those of mouse or other species. This in vitro model could be highly relevant in investigation of the mechanisms of trophoblast differentiation and its perturbations, as well as the trophoblast function in physiological and pathological conditions [134].

## Appreciating the epigenetic impact of the use of organoids to generate gametes and embryos

Assisted reproduction technologies (ART) have raised questions about their potential impact on epigenetic programming and the long-term consequences on the offspring health [164]. Here we have described additional approaches to generate gametes using organoids. During gametogenesis, large epigenome modifications occur in a coordinated way [165]. How the in vitro environment affects these processes has to be carefully evaluated. In the oocyte, from a large body of evidence DNA methylation appears robust and little affected by follicle culture from the preantral stage onwards. Genome wide analysis of methylation in mouse MII oocytes showed a globally conserved pattern in oocytes derived from in vitro developed follicles as compared to their in vivo ovulated counterparts [166]. GV ewe oocytes obtained after in vitro or in vivo follicle growth also displayed a similar level of global methylation [36]. In both studies, and in in vitro matured bovine oocytes [167], differentially methylated regions of imprinted genes appeared adequately methylated or unmethylated for later embryonic maternal or paternal allele specific expression, respectively. Following in vitro fertilization, the developmental rate to blastocyst was lower from mouse oocytes obtained after follicle culture than after superovulation, but the level of alteration in parental imprinting maintenance in both types of embryos was similar relative to their counterparts produced from non-stimulated females [168]. Overall, the methylome is globally reproduced in in vitro grown oocytes, and parental imprint adequately maintained in successfully developed blastocysts. However, outside this global pattern conservation, follicle culture and/or in vitro maturation are associated with alterations at few specific loci, as are other ART procedure such as ovarian stimulation [166]. Concerning spermatozoa, aberrant DNA methylation was present at some specific genomic loci in germline stem–like cells generated from mouse ESCs [169]. Despite these abnormalities, the spermatozoa derived from these germ cells were fertile, but potential long-term effects have not been evaluated. The generation of embryoids are very new and no studies have yet examined their epigenome. However, as ESCs are prone to loose imprinting upon prolonged culture, careful examination of the imprint status in the embryoids will be of major importance [170, 171]. On the other hand, embryo culture has been recognised as a major factor contributing to adverse effects of ART onto placental function [172]. In this regard, the use of oviduct and endometrium organoids may help to design conditions that would better meet the embryo needs.

## Conclusions

Organoids in the field of reproduction for farm animals constitute new highly valuable models but are still in their infancy. In a virtuous circle, progresses both necessitate and allow increasing our knowledge on the regulation of homeostasis in reproductive organs and embryonic development. Ultimately, the most exciting challenge will be to understand the interactions between somatic and germinal/embryonic cells leading to the success of reproduction by co-culturing organoids of different origins: oviduct organoid with spermatozoa or early embryos, possibly generated using gonadal organoids; or endometrium organoid with blastocyst/blastoid or trophoblast organoid. This collective work on stem cell physiology and cell culture also generates new stimulating interactions between researchers of various scientific fields.

## Data Availability

Not applicable.

## Abbreviations

2D: Two-dimensional
3D: Three-dimensional
ART: Assisted reproduction technologies
ASC: Adult stem/progenitor cell
ESC: Embryonic stem cell
hTERT: Human Telomerase Reverse Transcriptase
iPSC: Induced pluripotent stem cells
MSC: Mesenchymal stem cell
OEC: Oviduct epithelial cell
PGC: Primordial germ cell
SSC: Spermatogonial stem cell
TSC: Trophoblast stem cell
